# An Eco-Friendly Disposable Plasmonic Biochip Based on Bacterial Cellulose for Interleukin-17A Detection at Atto-Femto Molar Level

**DOI:** 10.1021/acsomega.5c10169

**Published:** 2026-02-23

**Authors:** Rosalba Pitruzzella, Chiara Marzano, Francesco Arcadio, Luigi Zeni, Salvatore Graziani, Carlo Trigona, Giovanna Di Pasquale, Antonino Pollicino, Nunzio Cennamo

**Affiliations:** † Department of Engineering, 18994 University of Campania Luigi Vanvitelli, Via Roma 29, Aversa 81031, Italy; ‡ Department of Electrical, Electronics and Computer Engineering (DIEEI), 9298 University of Catania, Viale Andrea Doria 6, Catania 95125, Italy; § Department of Chemical Sciences, University of Catania, Viale Andrea Doria 6, Catania 95125, Italy; ∥ Department of Civil Engineering and Architecture, University of Catania, Viale Andrea Doria 6, Catania 95125, Italy

## Abstract

The need for disposable, low-cost, biodegradable, small-size, and biocompatible sensor chips is crucial for the development of point-of-care tests (POCTs) in several bio/chemical sensing application fields. In this scenario, an eco-friendly sensor chip based on gold-coated bacterial cellulose (BC) nanostructures, which exploits localized surface plasmon resonance (LSPR) phenomena, is combined with a bioreceptor layer as a proof-of-concept for cytokine detection. The BC-based LSPR chip was developed and tested using a simple transmission-based experimental setup, enabling both bulk and binding sensitivity characterization. In particular, the optical analysis revealed a BC-based LSPR chip’s bulk sensitivity of about 370 nm/RIU, comparable to that of more complex and expensive LSPR platforms reported in the literature. Following a functionalization process with an antibody specific for the interleukin 17A (IL-17A) protein, the BC-based LSPR biochip was tested, demonstrating an ultralow detection limit in the atto-femto molar range (detection limit of approximately 400 aM), an ultrawide detection range, and good selectivity toward other interleukins, making it a proof-of-principle useful in biomedical applications. The achieved results paved the way for the applicability of this eco-friendly biochip as a disposable chip useful for POCTs in several application fields, where the required disposable feature is due to contamination that occurs during measurements in real-world scenarios.

## Graphical abstract

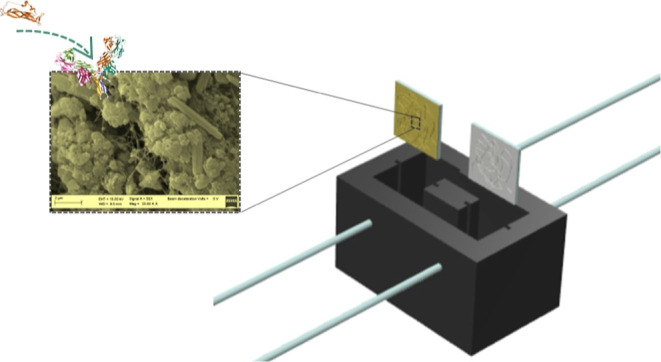

## 1 Introduction

In the field of biosensing, the demand for biocompatible, biodegradable, and disposable chips is becoming increasingly crucial. The present work, which exploits an eco-friendly disposable biochip, could be useful in addressing several aspects related to the environmental problems caused by the massive amount and indiscriminate use of nonbiodegradable medical and electronic waste, a concern that has been a pressing issue for the scientific community.^1–3^

During this time, there is a growing need for reliable, rapid tests that can also be used directly by anyone near the patient. In the context of point-of-care tests (POCTs), which are intended as tools for decentralized analysis, these aspects are becoming a key task to accomplish.^4,5^ POCT devices support applications in various domains, including food safety, environmental monitoring, and medical diagnostics. The capacity to monitor many analytes while maintaining the same measurement setup, together with the ability to perform measurements and store the data in the cloud automatically, is remarkably boosting their development.^6–9^

In this frame, in order to monitor analyte/receptor binding, plasmonic phenomena, meaning surface plasmon resonance (SPR), localized SPR (LSPR), and hybrid modes are frequently exploited as physical operating principles behind the operation of POCT devices.^10–12^ These techniques are extremely sensitive to refractive index (RI) variations at the interface between a metallic nanofilm and a dielectric medium. More specifically, SPR is excited by a continuous metallic nanofilm, whereas LSPR is generated from metallic nanoparticles or nanostructures.^13,14^ Alternatively, under certain conditions, both SPR and LSPR coexist, giving rise to plasmonic hybrid modes.^15^ Regarding POCT devices based on plasmonic phenomena, the use of “green” materials, which are intended to be biodegradable and biocompatible, is rapidly spreading due to the aforementioned reasons. To this purpose, an innovative approach that utilizes the natural nanostructures to realize nanoplasmonic platforms was recently proposed by Cennamo et al.^16^ In particular, by harnessing natural sunflower pollen nanotips, an ultralow limit of detection (less than one attomolar) was obtained in estradiol detection. Similarly, LSPR probes based on synthetic nanostructures, known as nanourchins, could represent a valid alternative,^17^ although their biocompatibility has not yet been fully assessed.^18^ Moreover, interesting LSPR WaveFlex biosensors were recently designed and fabricated. Specifically, plasmonic probes based on flexible W-shaped optical fibers are coupled with gold nanoparticles and other nanomaterials (zinc oxide nanoparticles, graphene oxide nanoparticles, cerium oxide nanorods, tungsten disulfide nanosheets, or multiwalled carbon nanotubes) to excite highly sensitive LSPR phenomena.^19–21^

An additional option for developing green plasmonic biosensors involves the use of new materials, such as biodegradable polymers.^22^ In this context, Hsuan-Pei E et al.^23^ recently proposed an SPR sensor based on optical fibers made of spider silk, which is a naturally protein-based biopolymer with great flexibility and low attenuation. Along the same line, Lee et al.^24^ developed an LSPR nanoplasmonic probe based on a 2D-gold disk array and silk, which was used as a glucose sensor in a proof-of-concept application. In addition, bacterial cellulose (BC) was recently demonstrated to fit this scope, being biocompatible and fully biodegradable, since it is realized by bacteria through a green process.^25^ By exploiting the nanofibers present in BC films and depositing a gold nanofilm onto the surface of a BC-based slab waveguide, innovative LSPR sensors were investigated.^26,27^

In this work, the BC-based plasmonic sensor is tested differently with respect to^26,27^ in order to excite the LSPR phenomenon more efficiently. More specifically, in this case, the BC is considered as a substrate rather than a slab waveguide propagating the light, able to excite LSPR phenomena. For this purpose, a transmission-based experimental setup similar to that described in^16^ was employed. The experimental setup is based on two plastic optical fibers (POFs) exploited to connect the white light source and the spectrometers with the BC chips.^16^ In the first step, optical tests were performed to obtain the bulk sensitivity of the plasmonic probe. Then, for the first time, the BC gold surface was functionalized with an antibody in order to obtain a biosensor chip. In particular, an antibody specific for interleukin 17A (IL-17A) has been used in this work. The analyte IL-17A is secreted by Th17 cells and other immune cells, including innate lymphoid cells, and represents the major mediator of tissue inflammation in many autoimmune diseases.^28^ High levels of this cytokine are associated with several chronic inflammatory diseases. In this context, they are, therefore, a therapeutic target in the treatment of various autoimmune diseases, including psoriasis, rheumatoid arthritis, and multiple sclerosis (MS).^29^ Moreover, it has been increasingly recognized for its role in cancer progression. IL-17A significantly influences tumor growth and prognosis in several types of cancers, with an important role in immune modulation.^30^ IL-17A up-regulates the expression of several inflammation-related genes in the target cells (keratinocytes and fibroblasts), increasing the production of chemokines, cytokines, antimicrobial peptides, and other mediators that contribute to signs and symptoms of the disease.

The proposed BC-based LSPR biochip for IL-17A has been tested as a proof-of-concept in a phosphate-buffered saline (PBS) solution, as reported in the Results and Discussion section, covering an ultrawide detection range and achieving an ultralow detection limit (LOD). Furthermore, the possibility of using low-cost, disposable, and eco-friendly chips is a crucial aspect in POCTs, especially in applications where there is a high degree of contamination during the measurements. Moreover, selectivity tests with other types of interleukins have been performed to confirm the specificity interaction monitored by the proposed BC-based biosensor system.

## 2 Materials and Methods

### 2.1 Bio/Chemicals

Purified CBP-GS005 film of BC (average thickness 154 μm) was purchased from BioFaber (Italy) and used as received without further purification. Recombinant human IL-17A protein (ab282392) was purchased from Abcam (Cambridge, CB2 0AX, UK), together with polyclonal antibody anti-IL-17A (ab79056). Recombinant human interleukin 1β (IL-1β) protein (ab259387) and recombinant human interleukin 18 (IL-18) protein (ab316093) were purchased from Abcam. Ethanol, *N*-hydroxysuccinimide (NHS), *N*-(3-(dimethylamino)­propyl)-*N*′-ethylcarbodiimide hydrochloride (EDC), lipoic acid, ethanolamine (2-aminoethanol), and PBS were purchased from Merck KGaA (Darmstadt, Germany).

### 2.2 BC Characterization Method

Scanning electron microscopy (SEM) micrographs were obtained using a Cambridge 90 instrument equipped with an energy-dispersive X-ray (EDX) microanalysis system.

X-ray photoelectron spectroscopy (XPS) spectra were recorded with a VG Microtech spectrometer with a CLAMII analyzer. The X-ray source (Mg Ka, 1253.6 eV) worked at 200 kV and 10 mA at a pressure <2 × 10^–8^ Torr. Pass energy: 100 eV for wide scans and 50 eV for narrow scans. The spectra were recorded with taking-off angles of 45°. Binding energies were referenced to the C–H level at 285.0 eV.

Fourier transform infrared (FTIR) spectroscopy was conducted using a PerkinElmer Spectrum 100 spectrometer at room temperature (RT), covering the range from 4000 to 650 cm^–1^, with a resolution of 2.0 cm^–1^. A universal ATR sampling accessory was employed, allowing direct measurements of the samples without any prior treatment.

Thermogravimetric analyses (TGA) were performed using a Shimadzu DTG-60 instrument. TGA curves were recorded at a heating rate of 10 °C/min under a static air atmosphere, over a temperature range from 35 to 700 °C. The mass of the analyzed samples ranged between 8.0 mg and 11.0 mg.

### 2.3 BC-Based LSPR Sensor Chip

The production process of the BC-based LSPR chip includes only a gold deposition step. More specifically, a 60 nm thick gold nanofilm is sputtered onto the BC-based substrate, which has a thickness of 154 μm, using a sputter coater machine (Safematic CCU-010, Zizers, Switzerland). The sputtering process was repeated three times with a current of 60 mA for 23 s (20 nm per step) at a pressure of 0.01 mbar.

### 2.4 Functionalization Protocol for the BC-Based LSPR Chips

The BC surface functionalization process was achieved following the protocol reported in^31^ and outlined in Figure 1. At first, the BC surface was washed three times with filtered Milli-Q water. Subsequently, lipoic acid (concentration of 0.3 mM in an 8% ethanol solution) was incubated on the gold surface overnight to expose carboxylic groups; then, activation was achieved by incubating for 20 min at RT with an EDC/NHS solution (200 mM/50 mM in PBS, pH 7.4). Next, the sensitive surface was washed three times with PBS, and then the anti-IL-17A antibody (0.5 mg/mL) was immobilized by incubating 20 μL of the solution for 2 h at RT. After performing three wash steps with PBS to remove anything noncovalently bound, ethanolamine (1 M concentration, pH 8.0) was incubated for 30 min at RT to block unreacted sites. Finally, the so-prepared BC-based LSPR biochip was stored overnight at 4 °C before using it.

**Figure 1:**
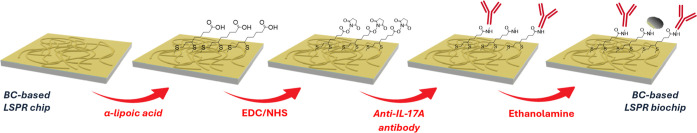
Outline of the functionalization steps performed on the BC-based LSPR chip.

### 2.5 Experimental Setup

The transmission-based experimental setup adopted to test the BC-based LSPR probe was the same one used in.^16^ Concisely, a broad-spectrum white light source (HL2000-LL, Ocean Insight, Orlando, FL, USA) is connected, through an optical splitter (50:50) made of plastic optical fibers (POFs), to two POF patches (980 μm PMMA core and 10 μm fluorinated cladding, with a total diameter of 1 mm). The two POFs launch the light toward the reference chip (the same BC film without gold) and the BC-based LSPR chip, both of which have dimensions of 10 mm × 10 mm. Both reference and plasmonic chips are housed in a custom holder realized by a 3D printer, which incorporates a measuring cell to host the liquid solution under test.

The POFs are located in the middle of the chips, similar to other nanoplasmonic chips.^16^

In order to easily accommodate both the BC-based plasmonic and reference chips, they were glued, along their borders, to 10 mm × 10 mm poly­(methyl methacrylate) (PMMA) substrates. On the opposite side, two analogous POF patches collect the transmitted light from both chips and direct it to two spectrometers (FLAME-S-VIR-NIR-ES, Ocean Insight, Orlando, FL, USA). Both the BC-based LSPR chip and the reference chip are orthogonally placed with respect to the input/output POF direction. Figure 2 reports an outline of the experimental setup described above.

**Figure 2:**
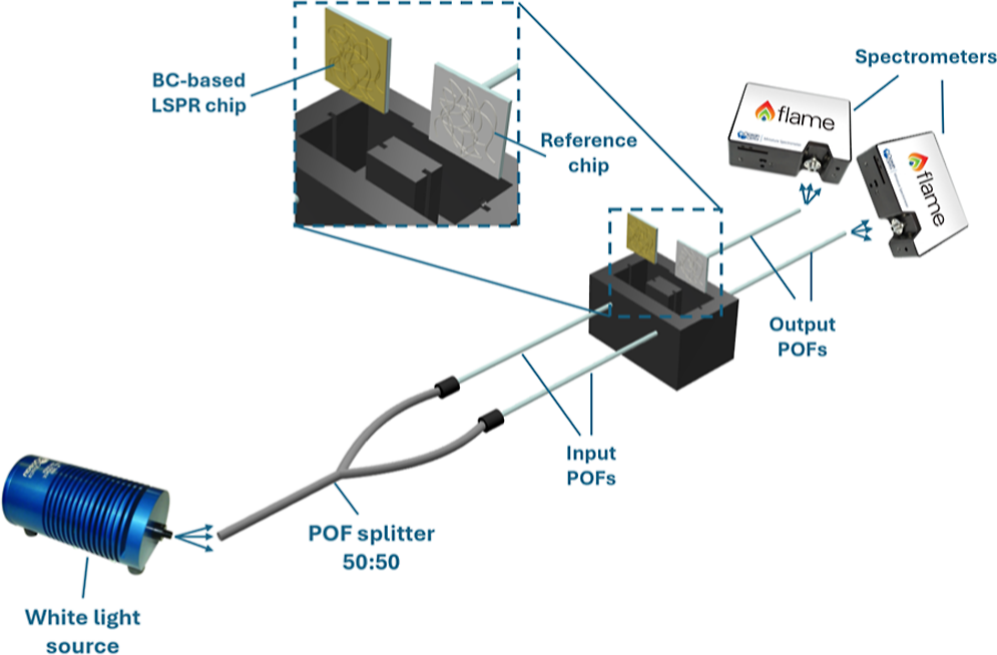
Outline of the experimental setup used to test the BC-based LSPR chip.

The experimental plasmonic spectra are obtained through a normalization process between the transmitted spectra achieved using the BC-based LSPR chip and the reference one.

### 2.6 Measurement Protocols

The BC-based LSPR chip was optically characterized by using water/glycerin mixtures having RIs ranging from 1.332 (water) to 1.362, previously checked by a commercial Abbe refractometer (RMI, Exacta + Optech GmbH, Munich, Germany). The optical tests of the bare LSPR surface were conducted by filling the measuring cell with 4 mL of the solution at different RI in order to obtain the bulk sensitivity.

Regarding the binding tests, after the functionalization step of the LSPR chip, the functionalized BC-based LSPR chips were tested with IL-17A solutions at increasing concentrations, ranging from 100 aM to 10 pM, prepared by serial dilution with PBS from the stock solution (concentration equal to 1 μM). Each IL-17A solution (4 mL volume) filling the measuring cell was incubated for 5 min. Next, washing steps with PBS were performed, and, finally, the spectrum was acquired by considering PBS as the bulk solution inside the measuring cell. All the plasmonic spectra were obtained using a normalization process performed by dividing the spectra transmitted by a BC-based LSPR chip by those transmitted by the reference chip (BC without gold). More specifically, after performing the normalization process using Matlab software (version R2022b, Mathworks, Natick, MA, USA), the LSPR spectra were smoothed using a Matlab “smooth” function (smoothing factor equal to 120) and then translated using pure translation along the *y*-axis to better compare the resonance values of the spectra. Finally, a standard MATLAB function can be used to determine the maximum values of the spectra.

## 3 Results and Discussion

### 3.1 BC Characterization

The SEM image (Figure 3) shows, besides nanofibers, an irregular surface with the presence of aggregates that could indicate impurities. The fiber arrangement shows a heterogeneous structure due to a nonuniform deposition and a possible modification of the drying process that altered the microstructure.

**Figure 3:**
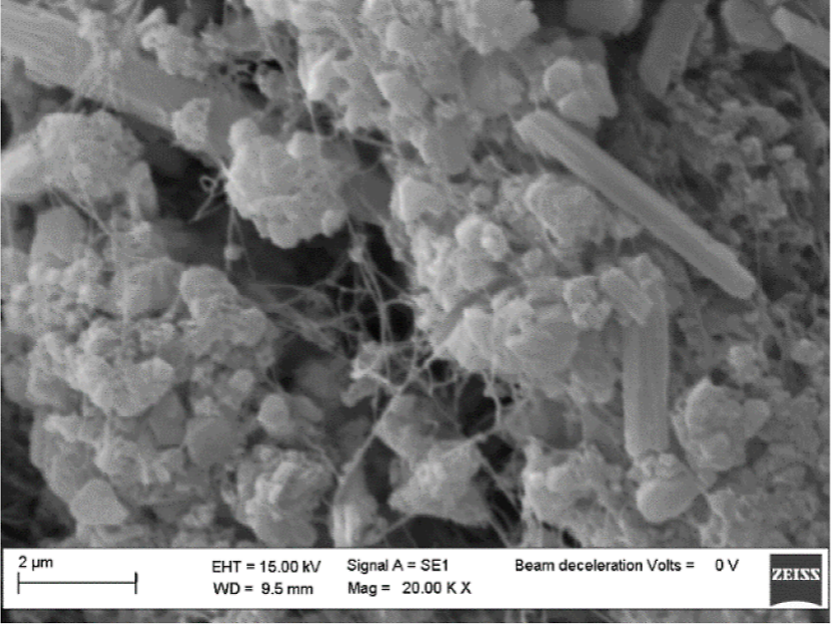
SEM image of the used BC.

An XPS analysis was conducted to determine the nature of any contaminants present on the outermost layers of the BC samples’ surface. The spectrum recorded over a wide range of binding energy is shown in Figure 4a. The minor amounts of silicon, sodium, nitrogen, calcium, aluminum, and chlorine detected in the wide scan (all below 1.5%) originate from the BC manufacturing and drying process: these elements are known residues from the fermentation medium and washing buffers, and are frequently reported in BC-based materials.^32–34^ Nevertheless, their presence is negligible and does not affect the optical properties of the gold-coated BC surface. To further investigate the chemical environment of carbon and oxygen, high-resolution XPS spectra were acquired for the C_1s_ and O_1s_ regions, as reported in Figure 4b,c. The C_1s_ spectrum displays a dominant peak at ∼287.0 eV, associated with C–O/C–OH bonds typical of cellulose (286.7 eV) along with higher binding energy components ascribable to O–C–O groups (288.1 eV). The O_1s_ signal is centered around 533.2 eV, confirming the prevalence of oxygen functionalities belonging to hydroxyl (532.9 eV) and glycosidic moieties (533.5 eV).

**Figure 4:**
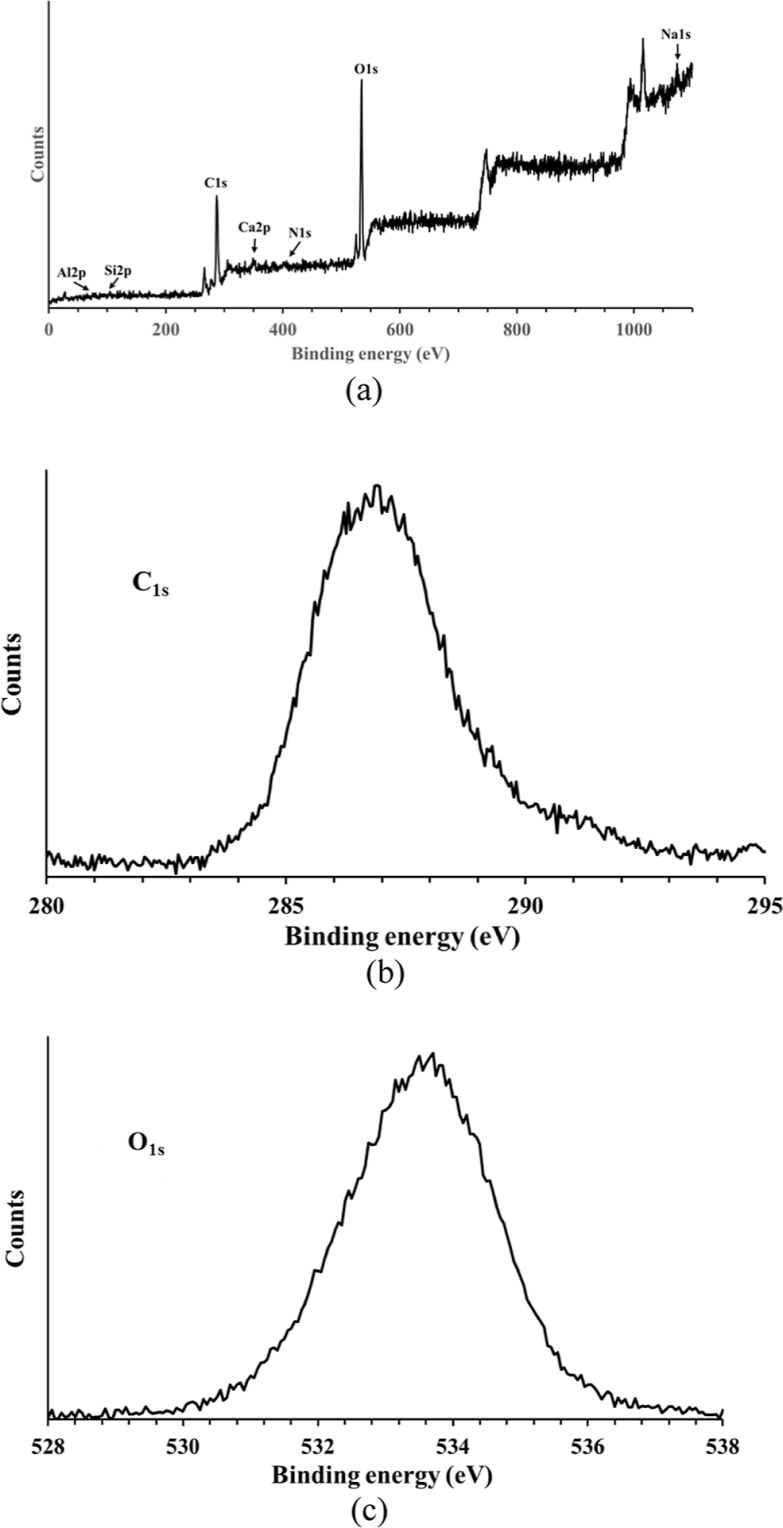
(a) XPS WideScan spectrum of BC sample. (b) High-resolution XPS scan of the C_1s_ region of the BC surface. (c) High-resolution XPS scan of the O_1s_ region of the BC surface.

The low-level contaminants observed in the wide scan, therefore, do not substantially influence the BC chemical surface composition, which remains largely governed by polysaccharide-derived species. The elemental analysis revealed the presence of contaminants, including silicon, sodium, nitrogen, calcium, aluminum, and chlorine, alongside carbon and oxygen from the cellulose. These contaminants were detected in amounts not exceeding 1.5%, with a total atomic abundance percentage of 7.5%. Bulk characterization was performed using ATR-IR and TGA analyses.

The ATR-FTIR spectrum of BC (see Figure 5) exhibits several characteristic absorption bands, confirming the presence of key functional groups typical of cellulose structures. A broad and intense absorption band is observed around 3449 cm^–1^, which corresponds to the O–H stretching vibration. This band is indicative of hydroxyl groups and highlights the extensive hydrogen bonding network present in the cellulose structure. In the region between 2800 cm^–1^ and 3000 cm^–1^, multiple peaks appear, including those at 2988 cm^–1^, 2919 cm^–1^, and 2800 cm^–1^, which correspond to C–H stretching vibrations of aliphatic groups. These signals are typically associated with the CH and CH_2_ groups present in the cellulose backbone. A distinct peak at 1741 cm^–1^ suggests the presence of C=O stretching vibrations, which may be attributed to carbonyl or carboxyl groups. This could indicate minor oxidation or residual functional groups from precursors used in BC production. The absorption band at 1384 cm^–1^ corresponds to C–H bending vibrations, which are associated with CH_2_ and CH_3_ groups. This peak further supports the presence of typical cellulose backbone structures. Another important absorption is found at 1175 cm^–1^, which is linked to C–O–C stretching vibrations of the glycosidic bond between glucose units. This peak is a key marker of cellulose, as it confirms the polymeric linkage characteristic of the material. Overall, the ATR-FTIR spectrum confirms the structural integrity of BC, with well-defined peaks corresponding to hydroxyl, aliphatic C–H, carbonyl, and glycosidic linkages. The presence of these characteristic bands supports the conclusion that the analyzed sample is predominantly composed of BC, with possible minor modifications resulting from processing conditions.

**Figure 5:**
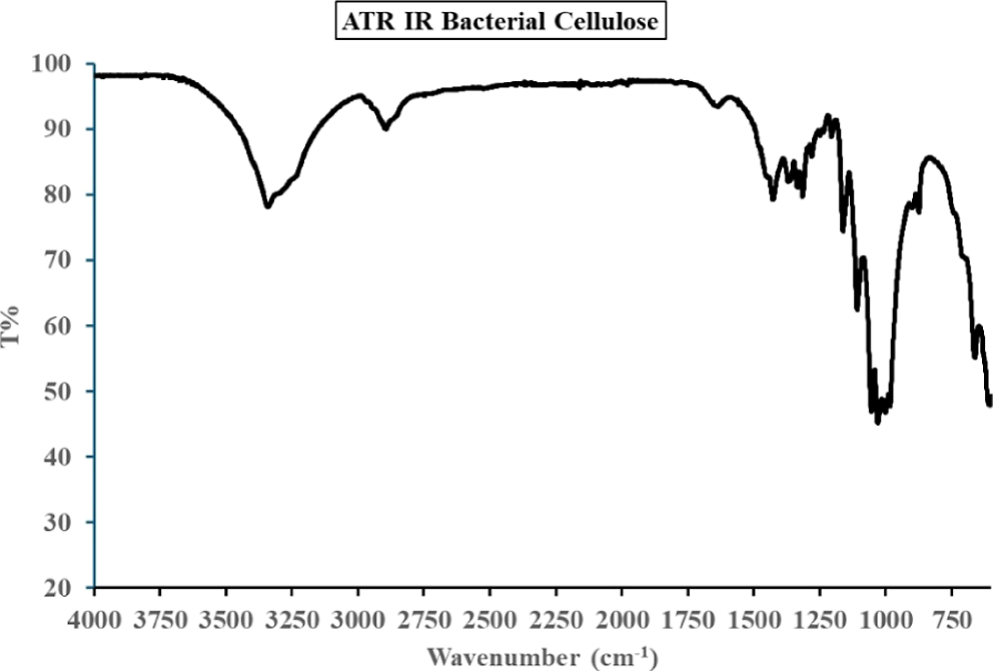
ATR-IR spectrum of BC sample.

The thermogravimetric analysis (TGA) of BC, reported in Figure 6, reveals its thermal stability and degradation behavior under controlled heating conditions. At lower temperatures, the sample exhibits some weight loss, indicating good thermal stability in this range. The mass reduction in this stage is typically attributed to the evaporation of absorbed water and other residual volatile compounds. Since BC is highly hydrophilic, some moisture retention is expected even after drying. A significant weight loss occurs around 245 °C, marking the onset of the main degradation phase. This stage corresponds to the thermal decomposition of cellulose polymer chains, particularly the breakdown of glycosidic bonds and depolymerization of cellulose into volatile compounds, such as levoglucosan, CO_2_, CO, and other small organic molecules. This primary degradation phase is characteristic of pure cellulose materials, typically occurring between 200 and 400 °C, with the highest weight loss rate near 325 °C. Beyond 400 °C, a secondary weight loss occurs, though it is less pronounced. This phase is associated with the slow decomposition of the remaining carbonaceous char, characterized by the highest weight loss rate at approximately 430 °C. At around 470 °C, the sample stabilizes with little further weight loss, indicating the presence of a residual fraction, likely to consist of thermally resistant carbon structures. The TGA results confirm that BC undergoes a two-step thermal degradation process: initial water loss followed by significant polymer decomposition, leaving behind a small residual char. The onset of degradation at ∼245 °C aligns well with the expected behavior of almost pure cellulose, confirming the material’s thermal stability and composition.

**Figure 6:**
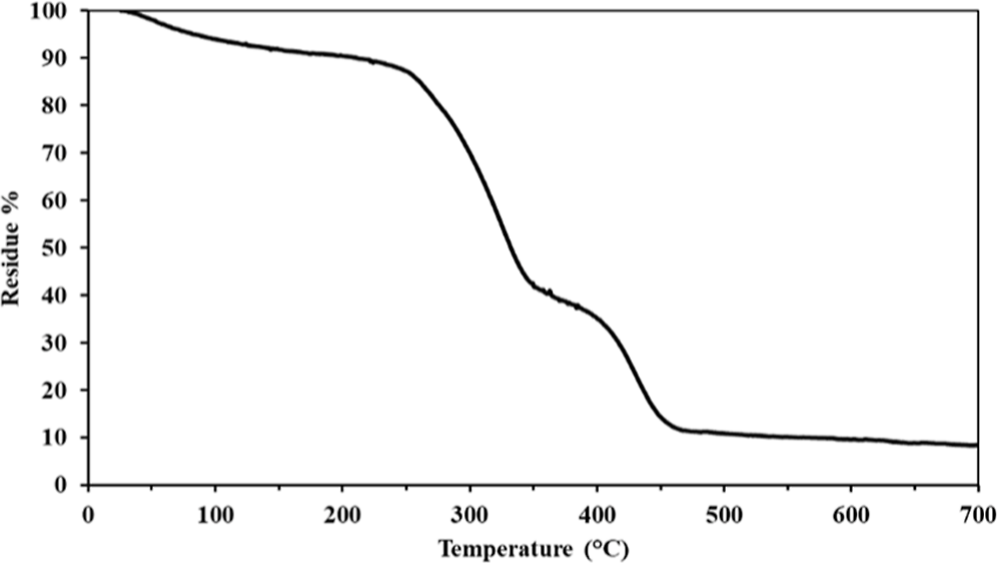
TGA thermogram of BC sample.

### 3.2 Optical Characterization: Bulk Sensitivity

Optical tests were performed by changing the RI of the solution filling the measuring cell to calculate the BC-based LSPR chip bulk sensitivity via the transmission-based experimental setup described in Section 2.5. As described in Section 2.6, four water/glycerin solutions with RIs of 1.332, 1.340, 1.353, and 1.362 were used to determine the optical response (in terms of bulk sensitivity). Five similar sensor chips have been tested using the same protocol to assess the reproducibility and repeatability of the BC-based LSPR chip. For instance, Figure 7a reports the plasmonic spectra achieved in air and water/glycerin solutions. Figure 7a shows that the plasmonic resonance wavelength shifts to lower values (blue-shift) when the bulk RI increases, in line with other nanoplasmonic sensors.^35^

**Figure 7:**
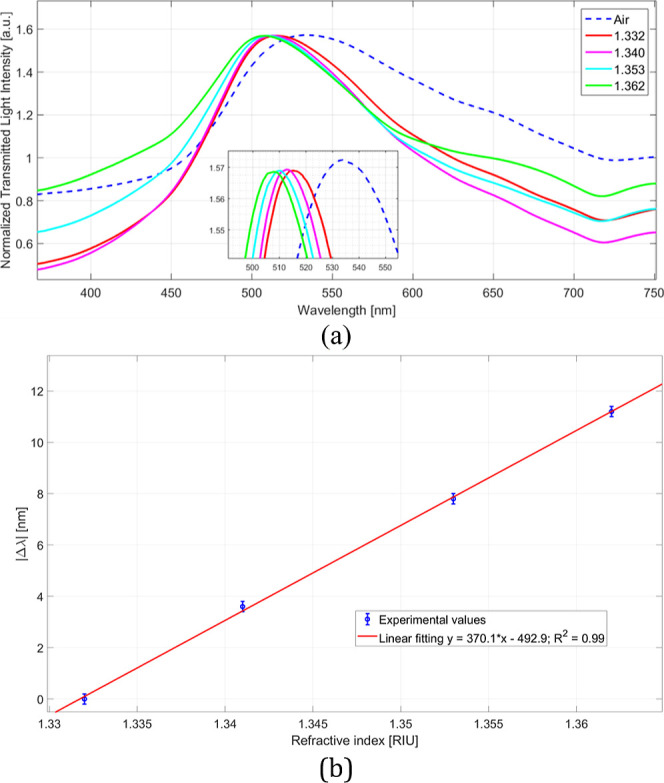
(a) Plasmonic spectra obtained by testing the BC-based LSPR chip with water/glycerin solutions at different RIs. (b) Absolute value of resonance wavelength variations (|Δλ|) calculated with respect to that of water (RI = 1.332) and linear fitting of the experimental values with relative error bar.

As shown in Figure 7a, this kind of plasmonic chip can also be used to detect analytes of interest in the aeriform matrix where the bulk RI is similar to the air (RI = 1). Figure 7b reports experimental data with the error bar in the aqueous matrix, i.e. the resonance wavelength shift in absolute value (|Δλ|) computed in relation to water (RI = 1.332), with the linear fitting of the values.

The error bar equal to 0.15 nm is the maximum standard deviation measured by repeating the same measurement on five similar chips in the same work condition. To evaluate the bulk sensitivity, eq 1 can be used

1 $$S=\frac{\delta \lambda }{\delta n}\left[nm/RIU\right]$$

where δλ represents the shift in plasmonic resonance wavelength obtained following a variation in the bulk solution RI equal to δn. By the linear fitting of Figure 7b and eq 1, the bulk sensitivity corresponds to the slope of the linear function, i.e. 370 nm/RIU.

This value is in line with those reported by other LSPR sensors presented in the literature, as shown in Table 1 for comparison.

**Table 1:** Comparison between Bulk Sensitivities of Several LSPR Sensor Configurations

LSPR sensor configuration	bulk sensitivity [nm/RIU]
2D array of silver nanoparticles^36^	357.14
core–shell structured gold nanocone array^37^	417
nanocavities-based^38^	405
BC-based chip	370

### 3.3 Binding Tests: IL-17A Detection

Three BC-based LSPR chips were functionalized following the functionalization procedure reported in Section 2.4 to determine the binding sensitivity of the IL-17A biosensor. To verify the success of the functionalization process described in Section 2.4, plasmonic spectra were acquired before and after the procedure using the same bulk solution, i.e., PBS. Figure 8 shows the resonance wavelength shifted to lower values (blue-shift).

**Figure 8:**
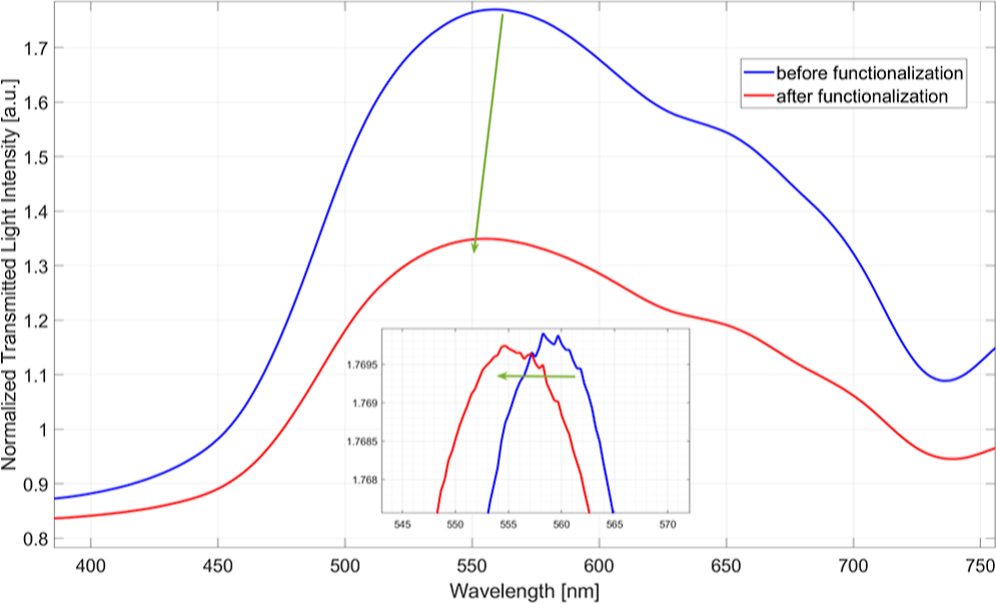
Plasmonic spectra achieved with PBS filling the measuring cell before (blue solid line) and after (red solid line) the functionalization process. The zoomed inset reports these spectra after an alignment procedure, so the shift in resonance wavelength can be better appreciated.

It is known that when the bioreceptor layer is present on the surface, the RI measured at the metal interface increases compared to the bare surface. Therefore, in line with the optical characterization results reported in Section 3.2, a blue-shift of the resonance wavelength was recorded. In particular, a shift of approximately 5 nm was obtained for all functionalized chips.

In a preliminary phase, to determine the optimal incubation time to ensure analyte–receptor interaction, the binding kinetics were studied. Figure 9 shows the plasmonic spectra obtained by monitoring the target analyte (IL-17A) at a concentration of 500 aM over time. Specifically, the specific analyte–receptor interaction was monitored for 5 min, acquiring the spectrum every minute (0, 1, 2, 3, 4, and 5 min).

**Figure 9:**
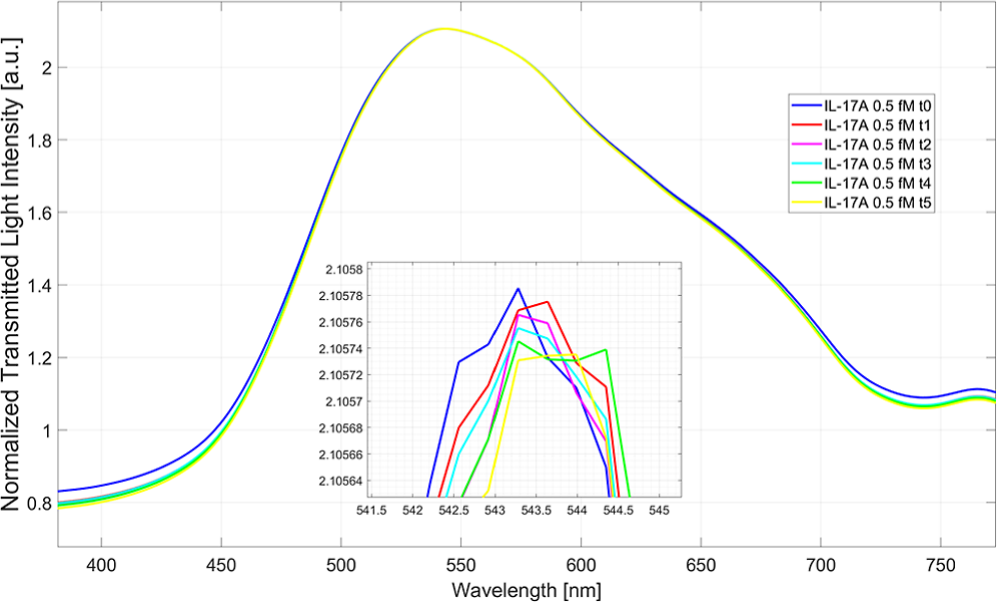
Plasmonic spectra achieved for IL-17A (500 aM) at different incubation times on the LSPR-BC biosensor. The spectra are acquired every minute for a total of 5 min.

As is evident in Figure 9, the optimal incubation time was reached at 4 min, since no variation in the resonance wavelength was observed after this time.

Next, to evaluate the BC-based LSPR biochips’ response to the analyte, the biosensor was tested with solutions containing IL-17A at different concentrations, as described in Section 2.6. In this frame, Figure 10 shows the plasmonic spectra obtained at increasing IL-17A concentrations, ranging from 0.1 fM to 10^4^ fM. As it is clear, when the binding between the bioreceptor layer and the IL-17A occurs, the measured refractive index decreases (fixed the bulk solution), so the resonance wavelength shifts to the right, similarly to what is obtained in.^31^

**Figure 10:**
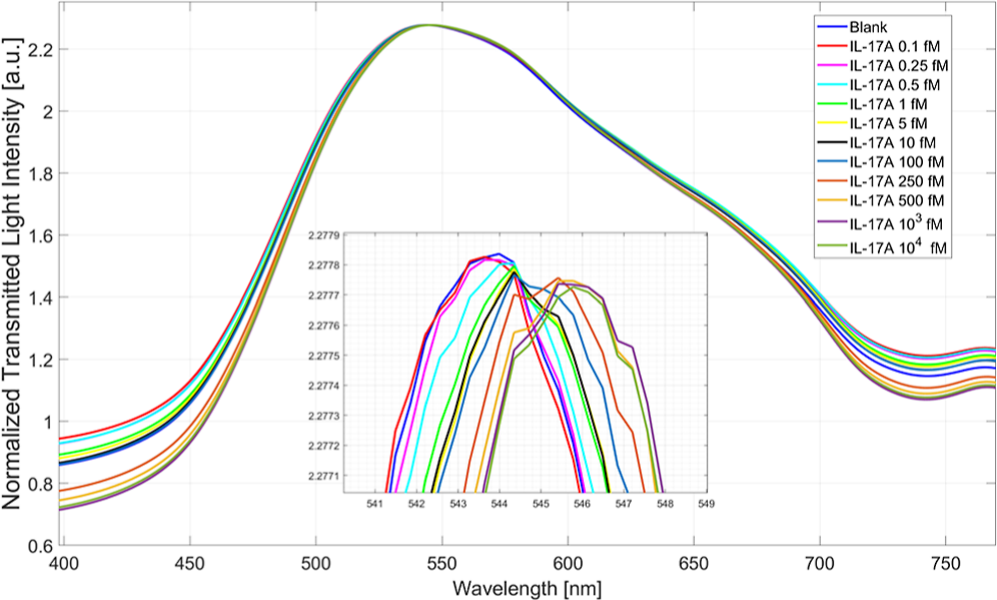
Plasmonic spectra achieved at different IL-17A concentrations (ranging from 0.1 fM to 10^4^ fM) in PBS.

It should be noted that the bioreceptor used is a polyclonal antibody; hence, several binding sites with different affinity constants are present, similar to.^39^ Consequently, two sites were tested, one occurring at low concentration values and another at higher concentrations. In this case, to describe this kind of interaction, the Bi-Langmuir model (see eq 2) is typically adopted.

2 $$\Delta \lambda_{c}=\Delta \lambda_{\max,1}\frac{c}{K_{1}+c}+\Delta \lambda_{\max,2}\frac{c}{K_{2}+c}$$

where *c* is the analyte concentration, Δλ_max_ corresponds to the maximum resonance wavelength shift with respect to the blank solution (Δλ_*c*_), reached at the saturation of the sites considered with the target analyte, and *K* is the dissociation constant of the target molecule for the recognition site considered. In particular, Δλ_max,1_ and K_1_ correspond to the parameters in relation to the sites with stronger affinity, while Δλ_max,2_ and *K*_2_ are relative to the sites with weaker affinity.

Figure 11 reports the Bi-Langmuir dose–response curve obtained by eq 2.

**Figure 11:**
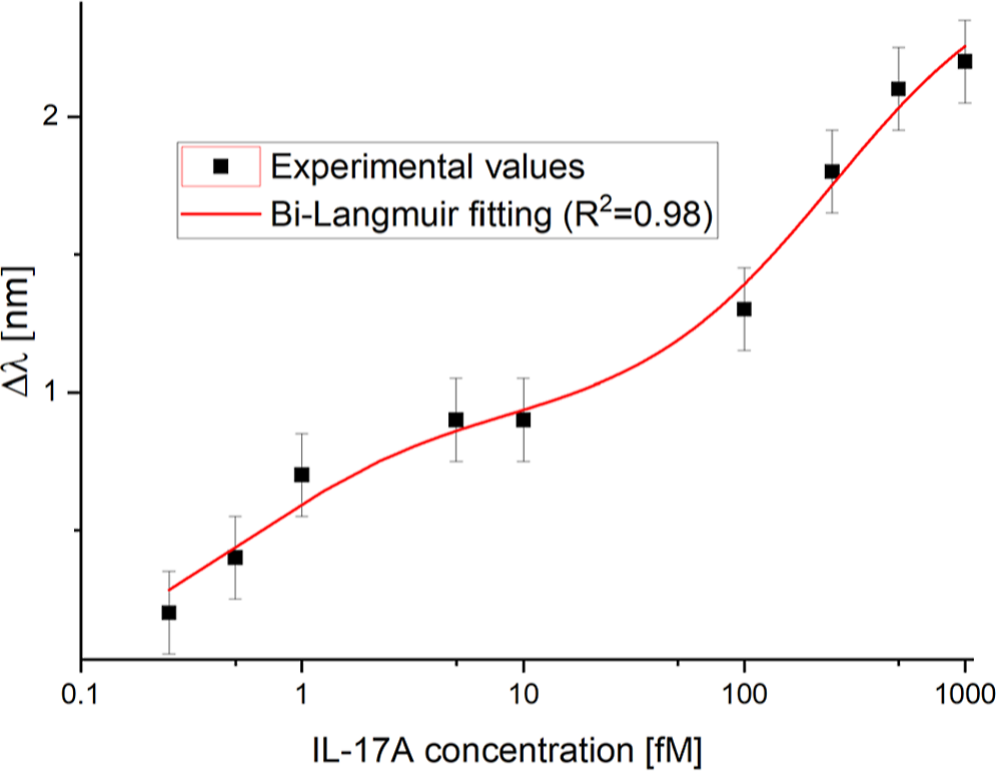
Resonance wavelength variations, calculated with respect to the blank, as a function of IL-17A concentration, and error bar. Experimental values were fitted by the Bi-Langmuir model.

The error bar reported in Figures 11 and 12 was calculated from the maximum standard deviation (the worst case) observed across three different BC-based LSPR biochips tested under similar conditions, resulting in 0.15 nm. This value is not used to estimate sensor performance. However, it is useful for assessing the quality of the model error, the standard deviation of the blank in the Langmuir fitting used to estimate the LOD.

**Figure 12:**
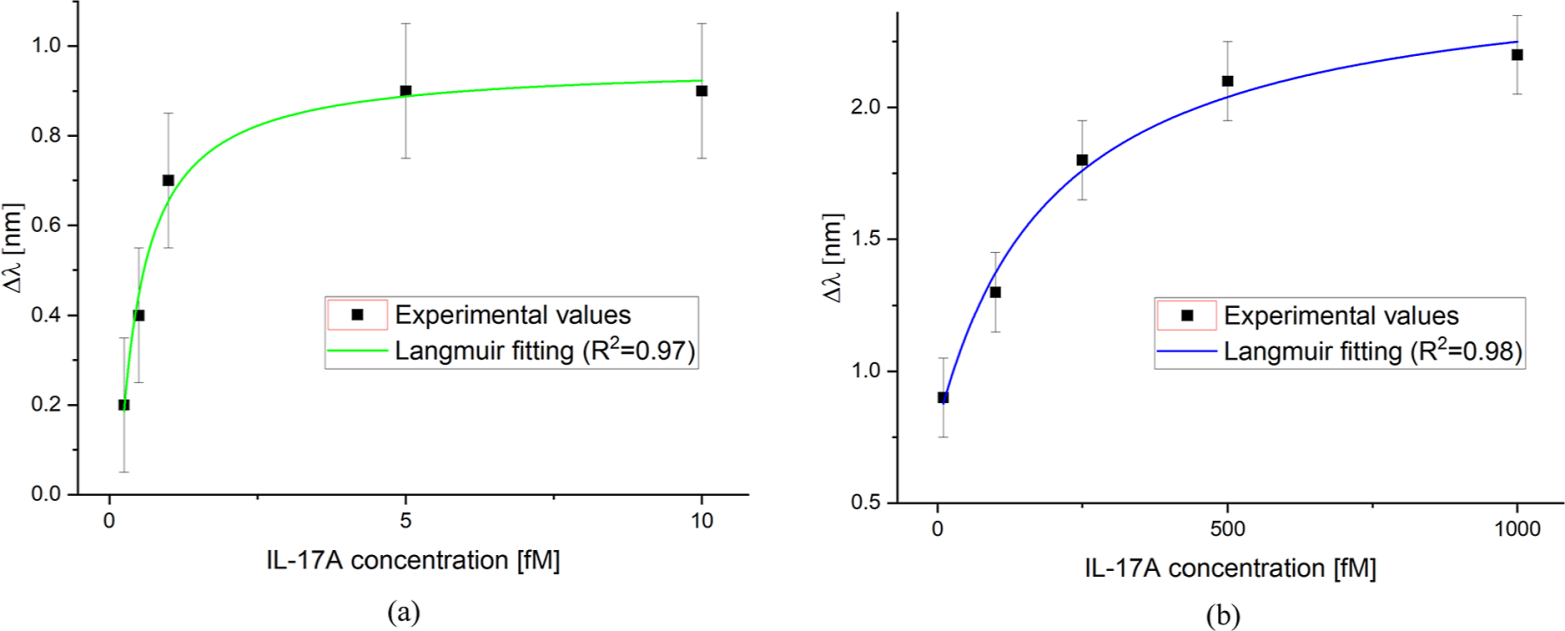
Dose–response curves achieved by Langmuir fitting in relation to (a) strong affinity and (b) weak affinity binding sites.

In order to calculate the biosensor performance parameters, only the low concentration range (relative to a stronger affinity) can be considered. At this end, one specific kind of site is considered and fitted using a Langmuir model, as reported in eq 3

3 $$\Delta \lambda_{c}=\lambda_{c}-\lambda_{0}=\Delta \lambda_{\max,i}\frac{c}{K_{i}+c}$$

where *i* is equal to 1 or 2 depending on whether the binding sites with the strongest or weakest affinity are considered, respectively.

Figure 12a reports the dose–response curve relative to the sites with stronger affinity (low concentration range). For the sake of completeness, a similar argument can be made with weaker affinity sites, whose dose–response curve is reported in Figure 12b. For both sites, Table 2 reports the Langmuir fitting parameters obtained by OriginPro software.

**Table 2:** Langmuir Fitting Parameters, Relative to Stronger and Weaker Binding Sites, for IL-17A Detection in PBS

binding sites	λ_0_ [nm]		Δλ_max, i_ [nm]		*K*_i_ [fM]		statistics	
	value	St. error	value	St. error	value	St. error	χ2	*R* ^2^
strong (*i* = 1)	–0.63	0.54	0.96	0.047	0.24	0.15	0.12	0.97
weak (*i* = 2)	0.79	0.09	2.55	0.16	202.82	67.01	0.3	0.98

By considering the relative parameters obtained via Langmuir fitting concerning the stronger binding sites, it is possible to evaluate the biosensor performance parameters, namely sensitivity at low concentration (*S*_lowc_), LOD, and the affinity constant (*K*_aff1_). More specifically, in the hypothesis that c≪K, eq 3 can be considered a linear function whose slope is defined as *S*_lowc_; the LOD is defined by the ratio between 3 times the standard deviation of the blank (st. error of λ_0_ in Table 2) and *S*_lowc_. For the affinity constants (*K*_aff1_ and *K*_aff2_), it is necessary to take the reciprocal of the *K*_*i*_ parameter for the stronger and weaker binding sites, respectively.^40^

In particular, relative to the stronger sites, a LOD of approximately 410 aM is obtained. On the other hand, the affinity constants resulted equal to 4.17 [fM]^−1^ and 0.005 [fM]^−1^ for the stronger and weaker sites, respectively. Table 3 summarizes the binding characteristic parameters of the BC-based LSPR biochip.

**Table 3:** BC-Based LSPR Biochip Binding Parameters for IL-17A Detection in PBS Solution

binding parameters	value
*S*_lowc_	4 [nm/fM]
LOD	0.4 [fM] (410 aM)
*K*_aff1_	4.17 [fM]^−1^
*K*_aff2_	0.005 [fM]^−1^
detection range	0.4–500 [fM]

The selectivity of the IL-17A biosensor was assessed by challenging the functionalized LSPR-BC probe with two other interleukins, namely IL-18 and IL-1β. The experimental results reported in Figure 13 show that the resonance wavelength shifts induced by the interfering substances (concentration of 1 nM) were negligible compared to those obtained with the analyte (IL-17A) at a concentration 6 orders of magnitude lower (1 fM). Therefore, even under these extreme conditions (high concentration values), the interferents produced a negligible response, whereas IL-17A at 1 fM produced a significant detectable resonance wavelength shift.

**Figure 13:**
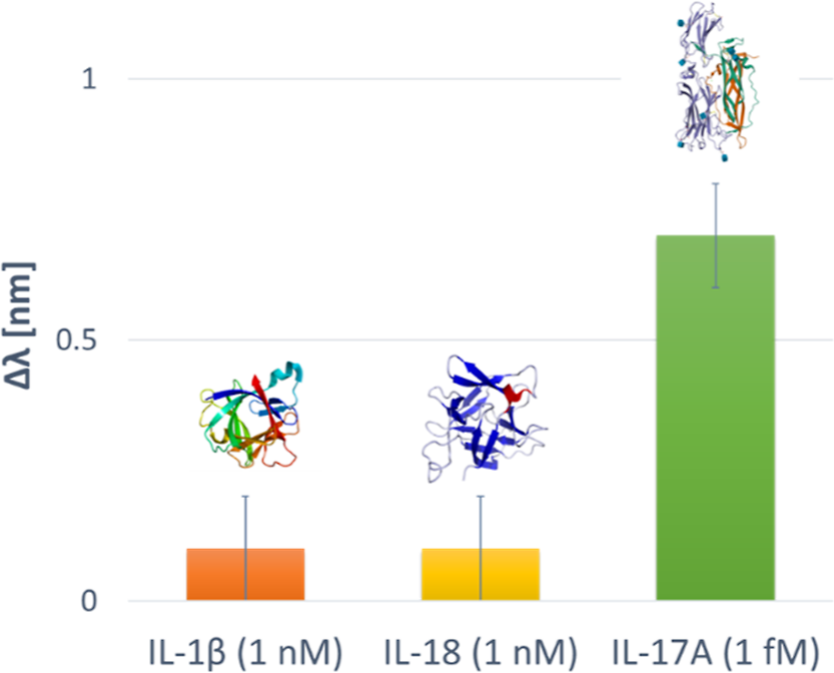
Comparison between the resonance wavelength variations obtained by testing the BC-based LSPR biochip with the interfering substances (IL-1β and IL-18) at 1 nM and the analyte target (IL-17A) at 1 fM.

Considering the results achieved through the proposed proof-of-concept in terms of both an ultralow detection limit and an ultrawide detection range, dilution steps of real samples can be utilized to detect the analyte in different real matrices, without nonspecific interaction with the sensitive surface.

To better contextualise the performance of the developed BC-based LSPR biosensor for detecting IL-17A, a two-way comparative analysis was conducted, as reported below.

The first comparative analysis, reported in Table 4, focuses on LSPR biosensors that utilize antibodies as recognition elements. This analysis summarizes key parameters such as sensor configuration, target analyte (including its molecular weight), LOD, and detection range, for several representative works present in the literature. This overview, as summarized in Table 4, enables a direct comparison of the proposed system with other LSPR biosensors in terms of analytical performance.

**Table 4:** Comparative Analysis of Antibody-Based LSPR Biosensors Reported in the Literature

biosensor configuration	analyte and molecular weight	LOD	detection range
LSPR-based PSA nanosensor^41^	prostate-specific antigen (PSA)	0.2 ng/mL (6.6 pM)	0.2–1 ng/mL (6.6–30.3 pM)
	33 kDa		
WaveFlex Biosensor^20^	aflatoxin B1 (AFB1)	7.12 nM	7.12 nM–100 nM
	312.27 Da		
sandwich-type LSPR sensor^42^	thyroglobulin (*T*_g_)	6.6 fg/mL (10 aM)	6.6 fg/mL–10 pg/mL (10 aM–15.15 fM)
	660 kDa		
LSPR-enhanced terahertz metasensors^43^	C-reactive protein (CRP)	100 pg/mL (0.87 pM)	100 pg/mL–100 μg/mL (0.87 pM–870 nM)
	115 kDa		
LSPR biosensor based on Au/PDA/AuNps comodified PCF^44^	rabbit immunoglobulin G(IgG)	0.021 μg/mL (140 pM)	0.021–20 μg/mL (140 pM–133 nM)
	150 kDa		
BC-based LSPR biochip	interleukin-17A	410 aM	0.4 fM–500 fM
	15 kDa		

The second comparison examines other biosensing approaches for detecting IL-17A based on several transduction mechanisms. Table 5 reports the detection principle, sample matrix tested, LOD, and detection range of each method. This broader comparison provides a comprehensive perspective on the state of the art in IL-17A detection, highlighting that the proposed LSPR-based strategy exhibits high sensitivity, almost comparable to that of other plasmonic approaches.

**Table 5:** Comparison of Biosensors for IL-17A Detection Using Different Transduction Methods

detection method	matrix	LOD	detection range
electrochemistry^45^	buffer	0.05 pg/mL (2.85 fM)	0.05 pg–1 ng/mL (2.85 fM–57 pM)
photoelectrochemical^46^	Human serum	<50 pg/mL (3.3 pM)	50–500 pg/mL (3.3 pM–33 pM)
hybrid plasmonic modes^47^	buffer	0.46 aM	0.1 aM–50 aM
multimode POFs and unconventional SPR^48^	buffer	4.2 aM	4.2 aM–5 fM
LSPR	buffer	410 aM	0.4 fM–500 fM

Based on the experimental results from the proposed proof-of-concept, future work could include detecting additional cytokines or other biomarkers using the same BC-based LSPR chip by simply modifying the specific antibody during functionalization. Another promising direction is the development of fully integrated systems by combining the BC-LSPR sensors with microfluidic and mechatronics systems, enabling automated sample handling and on-chip analysis.

## 4 Conclusions

An eco-friendly LSPR sensor chip based on bacterial cellulose (BC) was developed and investigated as a plasmonic platform using a simple transmission-based setup via POFs. In this work, the BC-based LSPR probe has been tested in a manner different from previous works.^26,27^ Optical characterization revealed a bulk sensitivity of 370 nm/RIU, which is comparable to that of state-of-the-art LSPR platforms. As a proof-of-concept, a bioreceptor layer combined with the BC-based LSPR probe was tested to demonstrate highly sensitive detection of IL-17A protein, achieving an ultralow limit of detection of 410 aM, together with an ultrawide detection range. Moreover, the developed biosensor system demonstrated excellent selectivity against potential interfering cytokines, such as IL-1β and IL-18. Overall, these experimental results confirm the capabilities of this sustainable and disposable BC-based LSPR sensor as a promising transducer chip to monitor receptor–analyte interactions, which are useful for analysis via POCTs. Considering the high performance in terms of detection limit and detection range, dilution steps can be exploited to enable analyte detection in several complex real matrices. In addition to its high sensitivity and specificity, another significant advantage of the proposed biosensor is its ability to perform analyses in a very short time (less than 5 min), making it suitable for monitoring molecules with short half-lives and for studying their dynamic secretion profiles.

Finally, the disposability of the proposed sensor is necessary to address contamination that can occur during measurements in real-world scenarios. Considering that the proposed sensing strategy can be used to monitor several substances of interest in real-world scenarios by changing the MREs combined with the plasmonic BC-based probe, such as for detecting analytes in agriculture, food, environmental monitoring, security, and biomedical applications. Therefore, the capability to use eco-friendly disposable chips is a key aspect in PoCTs because disposable sensor chips will be increasingly used in the future. Consequently, a plan for the disposal of disposable chips is necessary, and the capability to use green, biodegradable, and low-cost materials, such as BC-based chips, could be beneficial in this regard.

## References

[1] Sreejith S., Leo Joseph L. M. I., Kollem S., Vijumon V. T., Ajayan J. (2023). Biodegradable
sensors: A comprehensive review. Measurement.

[2] Hosseini E. S., Dervin S., Ganguly P., Dahiya R. (2021). Biodegradable materials
for sustainable health monitoring devices. ACS
Appl. Bio Mater..

[3] Chen X., Ahn J.-H. (2020). Biodegradable and
bioabsorbable sensors based on two-dimensional
materials. J. Mater. Chem. B.

[4] Ongaro A. E., Ndlovu Z., Sollier E., Otieno C., Ondoa P., Street A., Kersaudy-Kerhoas M. (2022). Engineering a sustainable future
for point-of-care diagnostics and single-use microfluidic devices. Lab Chip.

[5] Li P., Lee G.-H., Kim S. Y., Kwon S. Y., Kim H.-R., Park S. (2021). From diagnosis to treatment:
Recent advances in patient-friendly
biosensors and implantable devices. ACS Nano.

[6] Adedokun G., Alipanah M., Fan Z. H. (2024). Sample preparation
and detection
methods in point-of-care devices towards future at-home testing. Lab Chip.

[7] Rasheed S., Kanwal T., Ahmad N., Fatima B., Najam-ul-Haq M., Hussain D. (2024). Advances and challenges
in portable optical biosensors
for onsite detection and point-of-care diagnostics. TrAC Trends Anal. Chem..

[8] Khodaparast M., Sharley D., Marshall S., Beddoe T. (2024). Advances in point-of-care
and molecular techniques to detect waterborne pathogens. npj Clean Water.

[9] Shrivastava S., Trung T. Q., Lee N.-E. (2020). Recent
progress, challenges, and
prospects of fully integrated mobile and wearable point-of-care testing
systems for self-testing. Chem. Soc. Rev..

[10] Cennamo N., Pesavento M., Arcadio F., Marzano C., Zeni L. (2024). Advances in
plastic optical fiber bio/chemical sensors to realize point-of-care
tests. TrAC Trends Anal. Chem..

[11] Fan H., Li R., Chen Y., Da Q., Xiong C., Zhang Y., Qin Z., Liu G. L., Huang L. (2024). Sample-to-answer
point-of-care testing
platform for quantitative detection of small molecules in blood using
a smartphone- and microfluidic-based nanoplasmonic biosensor. Chem. Eng. J..

[12] Polonschii C., Rosu-Hamzescu M., David S., Oloumi A., Ursu V.-D., Szardenings M., Kern K., El Salhi A. E., Gheorghiu E. (2024). Point-of-care
personalized rapid diagnosis of allergies using peptide epitopes and
SPR multiplexed detection. Sens. Actuators B
Chem..

[13] Kumari A., Yadav A., Singh O. P., Sharan P. (2024). A review of surface
plasmon resonance (SPR) technology in biosensing: Innovations, applications
and future trends. J. Opt..

[14] Bhalla N., Shen A. Q. (2024). Localized surface
plasmon resonance sensing and its
interplay with fluidics. Langmuir.

[15] Dana B. D., Boyu J., Lin J., Li L., Koya A. N., Li W. (2023). Hybrid plasmonic modes for enhanced
refractive index sensing. Adv. Sens. Res..

[16] Cennamo N., Pasquardini L., Arcadio F., Zeni L. (2025). Pollen-based natural
nanostructures to realize nanoplasmonic biochips for single-molecule
detection. Sens. Actuators B Chem..

[17] Nair R. V., Yi P. J., Padmanabhan P., Gulyás B., Murukeshan V. M. (2020). Au nano-urchins enabled localized
surface plasmon resonance
sensing of beta amyloid fibrillation. Nanoscale
Adv..

[18] Ma R., Xiang L., Zhao X., Yin J. (2022). Progress in preparation
of sea urchin-like micro-/nanoparticles. Materials.

[19] Singh R., Zhang W., Liu X., Zhang B., Kumar S. (2023). Humanoid-shaped
WaveFlex biosensor for the detection of food contamination. Biomed. Opt. Express.

[20] Liu X., Singh R., Li G., Marques C., Zhang B., Kumar S. (2023). WaveFlex biosensor
using novel tri-tapered-in-tapered four-core fiber
with multimode fiber coupling for detection of aflatoxin B1. J. Lightwave Technol..

[21] Zhang G., Singh R., Zhang B., Kumar S., Li G. (2023). WaveFlex biosensor
based on S-tapered and waist-expanded technique for detection of glycosylated
hemoglobin. Biomed. Opt. Express.

[22] Koh L. M., Khor S. M. (2022). Current state and future prospects of sensors for evaluating
polymer biodegradability and sensors made from biodegradable polymers:
A review. Anal. Chim. Acta.

[23] E H.-P., Kong J. A. N., Chen W.-C., Chen C.-C., Cheng C.-H., Liu C.-Y. (2022). Biocompatible
spider silk-based metal–dielectric
fiber optic sugar sensor. Biomed. Opt. Express.

[24] Lee M., Jeon H., Kim S. (2015). A highly tunable and fully biocompatible
silk nanoplasmonic optical sensor. Nano Lett..

[25] Jeon J.-H., Oh I.-K., Kee C.-D., Kim S.-J. (2010). Bacterial cellulose
actuator with electrically driven bending deformation in hydrated
condition. Sens. Actuators B Chem..

[26] Cennamo N., Trigona C., Graziani S., Zeni L., Arcadio F., Di Pasquale G., Pollicino A. (2019). An eco-friendly disposable plasmonic
sensor based on bacterial cellulose and gold. Sensors.

[27] Cennamo N., Trigona C., Graziani S., Zeni L., Arcadio F., Xiaoyan L., Di Pasquale G., Pollicino A. (2021). Green LSPR
sensors based on thin bacterial cellulose waveguides for disposable
biosensor implementation. IEEE Trans. Instrum.
Meas..

[28] Kirkham B. W., Kavanaugh A., Reich K. (2014). Interleukin-17A: A unique pathway
in immune-mediated diseases: Psoriasis, psoriatic arthritis and rheumatoid
arthritis. Immunology.

[29] McGinley A. M., Sutton C. E., Edwards S. C., Leane C. M., DeCourcey J., Teijeiro A., Hamilton J. A., Boon L., Djouder N., Mills K. H. G. (2020). Interleukin-17A serves a priming role in autoimmunity
by recruiting IL-1β-producing myeloid cells that promote pathogenic
T cells. Immunity.

[30] Begagic E., Vranic S., Sominanda A. (2025). The role of
interleukin 17 in cancer:
a systematic review. Carcinogenesis.

[31] Cennamo N., Piccirillo A., Bencivenga D., Arcadio F., Annunziata M., Della Ragione F., Guida L., Zeni L., Borriello A. (2023). Towards a
point-of-care test to cover atto-femto and pico-nano molar concentration
ranges in interleukin-6 detection exploiting PMMA-based plasmonic
biosensor chips. Talanta.

[32] Pommet M., Juntaro J., Heng J. Y. Y., Mantalaris A., Lee A. F., Wilson K., Kalinka G., Shaffer M. S. P., Bismarck A. (2008). Surface modification of natural fibers using bacteria:
Depositing bacterial cellulose onto natural fibers to create hierarchical
fiber-reinforced nanocomposites. Biomacromolecules.

[33] Popa L., Ghica M. V., Tudoroiu E.-E., Ionescu D.-G., Dinu-Pîrvu C.-E. (2022). Bacterial
cellulose—A remarkable polymer as a source for biomaterials
tailoring. Materials.

[34] Dai Q., Bai Y., Fu B., Yang F. (2023). Multifunctional bacterial cellulose
films enabled by deep eutectic solvent-extracted lignin. ACS Omega.

[35] Zou W., Xie H., Ye Y., Ni W. (2019). Tailoring optical cross sections
of gold nanorods at a target plasmonic resonance wavelength using
bromosalicylic acid. RSC Adv..

[36] Willett D. R., Chumanov G. (2014). LSPR sensor combining sharp resonance and differential
optical measurements. Plasmonics.

[37] Kawasaki D., Yamada H., Maeno K., Sueyoshi K., Hisamoto H., Endo T. (2019). Core–shell-structured
gold nanocone array for label-free DNA
sensing. ACS Appl. Nano Mater..

[38] Cattoni A., Ghenuche P., Haghiri-Gosnet A.-M., Decanini D., Chen J., Pelouard J.-L., Collin S. (2011). λ3/1000
plasmonic nanocavities
for biosensing fabricated by soft UV nanoimprint lithography. Nano Lett..

[39] Bencivenga D., Arcadio F., Piccirillo A., Annunziata M., Della Ragione F., Cennamo N., Borriello A., Zeni L., Guida L. (2023). Plasmonic optical fiber biosensor
development for point-of-care detection of malondialdehyde as a biomarker
of oxidative stress. Free Radic. Biol. Med..

[40] Thompson M., Ellison S. L. R., Wood R. (2002). Harmonized
guidelines for single-laboratory
validation of methods of analysis (IUPAC technical report). Pure Appl. Chem..

[41] Mahani M., Alimohamadi F., Torkzadeh-Mahani M., Hassani Z., Khakbaz F., Divsar F., Yoosefian M. (2021). LSPR biosensing for early-stage prostate
cancer detection using hydrogen bonds between PSA and antibody: Molecular
dynamic and experimental study. J. Mol. Liq..

[42] Kim H.-M., Kim H.-J., Park J.-H., Lee S.-K. (2022). High-performance
biosensor using a sandwich assay via antibody-conjugated gold nanoparticles
and fiber-optic localized surface plasmon resonance. Anal. Chim. Acta.

[43] Wan M., Li Z., Chen Z., Liang L., Yan X., Yao H., Li Z., Wang Z., Hu X., Sun Z., Liang K., Wu G., Liu Q. (2025). LSPR-enhanced terahertz metasensors for highly sensitive
CRP detection via antibody functionalization. Preprint (SSRN).

[44] Wang Z., Zhang A., Chang P., Shi Y., Li Z. (2025). Sensitivity-enhanced
SPR/LSPR biosensor based on Au/PDA/AuNPs co-modified PCF for rabbit
IgG detection. Opt. Fiber Technol..

[45] Jiang W., Li T., Qu F., Ding L., Shen Z., Yang M. (2013). Electrochemical
immunosensor for the detection of interleukin-17 based on Cd2+-incorporated
polystyrene spheres. Sens. Actuators B Chem..

[46] Li X., Huang F., Bao C., Shao R., Deng L., Yang M. (2024). Development of photoelectrochemical
immunosensor based on halide
perovskite protected by organometallic compounds for determining interleukin-17A
(IL-17A). Microchim. Acta.

[47] Marzano C., Pitruzzella R., Arcadio F., Passeggio F., Seggio M., Zeni L., Pasquardini L., Cennamo N. (2025). Detecting attomolar concentrations of interleukin IL-17A
via pollen-based nanoplasmonic biochips. Biosensors.

[48] Cennamo N., Arcadio F., Marzano C., Pitruzzella R., Seggio M., Pesavento M., Toldo S., Abbate A., Zeni L. (2025). An attomolar-level optical device for monitoring receptor–analyte
interactions without functionalization steps: A case study of cytokine
detection. Sensors.

